# Correction: Microcultivation and FTIR spectroscopy-based screening revealed a nutrient-induced co-production of high-value metabolites in oleaginous *Mucoromycota* fungi

**DOI:** 10.1371/journal.pone.0245016

**Published:** 2020-12-31

**Authors:** The PLOS ONE Staff

## Notice of republication

An incorrect version of Figs 1 and 5 was published in error. The publisher apologizes for this error. This article was republished on Jun 26, 2020, to correct for this error. Please download this article again to view the correct version.

## Supporting information

S1 File Originally published, uncorrected article. (PDF)

S2 File Republished corrected article. (PDF)
