# Supplementary material for: Microcultivation and FTIR spectroscopy-based screening revealed a nutrient-induced co-production of high-value metabolites in oleaginous Mucoromycota fungi
Source: PLoS One. 2020 Jun 22;15(6):e0234870. doi: 10.1371/journal.pone.0234870 (PMC7307774; doi:10.1371/journal.pone.0234870)
Supplement: S13 Fig — (DOCX) [file pone.0234870.s013.docx]

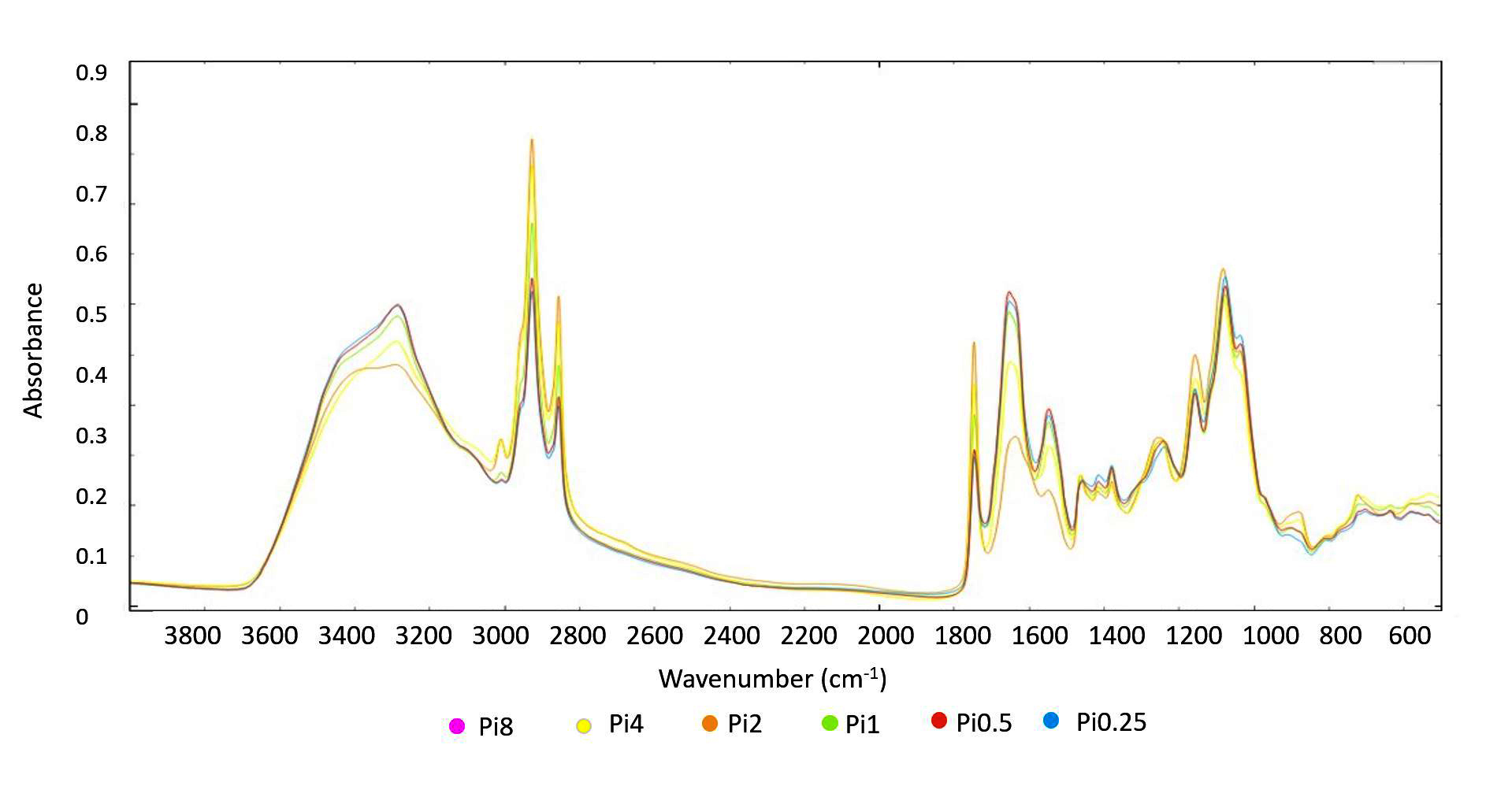


**Supplementary Figure 13.** FTIR-HTS spectra of *Mortierella hyalina* (EMSC corrected); ammonium sulphate nitrogen source, different Pi-levels
