# Supplementary material for: Microcultivation and FTIR spectroscopy-based screening revealed a nutrient-induced co-production of high-value metabolites in oleaginous Mucoromycota fungi
Source: PLoS One. 2020 Jun 22;15(6):e0234870. doi: 10.1371/journal.pone.0234870 (PMC7307774; doi:10.1371/journal.pone.0234870)
Supplement: S20 Fig — Titration of 100 ml not autoclaved YE-Pi0.25 (blue) and AS-Pi0.25 (red) with 1M HCl confirmed the buffering properties of yeast extract. (DOCX) [file pone.0234870.s020.docx]

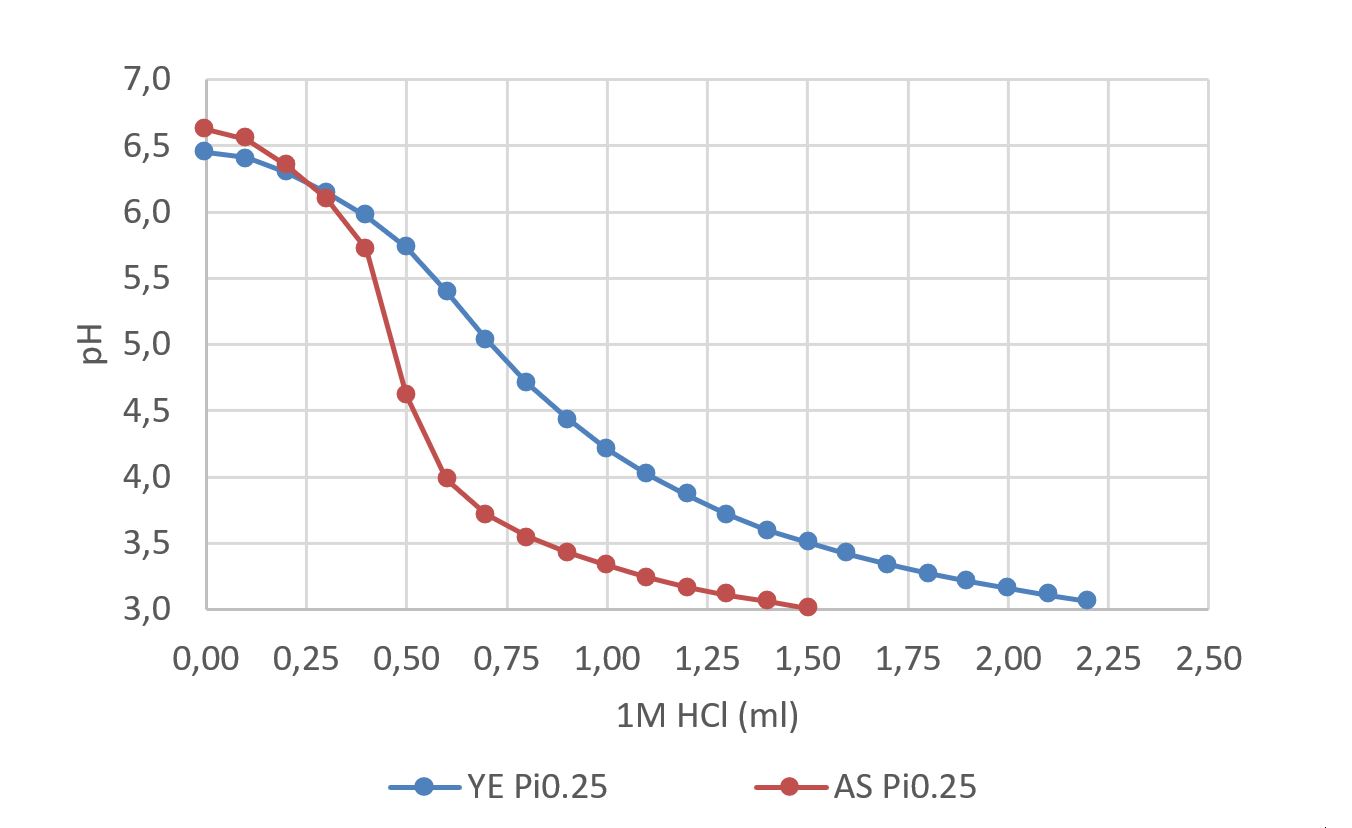


**Supplementary Figure 20.** Titration of 100 ml not autoclaved YE-Pi0.25 (blue) and AS-Pi0.25 (red) with 1M HCl confirmed the buffering properties of yeast extract.
